# The choose of different surgical therapies of hepatic alveolar echinococcosis: A single-center retrospective case-control study

**DOI:** 10.1097/MD.0000000000010033

**Published:** 2018-02-23

**Authors:** Ke-fei Chen, You-yin Tang, Rui Wang, Dan Fang, Jun-Hua Chen, Yong Zeng, Bo Li, Tian-fu Wen, Wen-tao Wang, Hong Wu, Ming-qing Xu, Jia-yin Yang, Yong-gang Wei, Ji-wei Huang, Jia-xin Li, Han-zhi Zhang, Xi Feng, Lü-nan Yan, Zhe-yu Chen

**Affiliations:** a Department of Liver Surgery, Liver Transplantation Center, West China Hospital of Sichuan University, Chengdu; b Institute of hydatid disease prevention and control, Ganze prefecture, Sichuan Province, China; c Molecular Pharmacology and Chemistry Program, Memorial Sloan-Kettering Cancer Center, New York, NY 10065.

**Keywords:** hepatic alveolar echinococcosis, liver transplantation, palliative hepatic resection, radical hepatic resection

## Abstract

The aim of this study was to evaluate different surgical therapies for hepatic alveolar echinococcosis in different clinical stages.

We analyze the clinical data of 115 patients who received surgical treatment in West China Hospital from January 2004 to June 2016. Among these patients, 77 cases underwent radical hepatic resection (group A, n = 77); 17 cases underwent palliative resection (group B, n = 17), and 21 cases underwent liver transplantation (group C, n = 21) with 12 cases of orthotopic liver transplantation and 9 cases of liver autotransplantation.

The postoperative complication rate of radical hepatic resection group was 13.0% (10/77), which is statistically significant (*P < .*05) than the rate of palliative resection group 29.4% (5/17) or liver transplantation group 23.8% (5/21). The follow-up period ranged from 1 to 72 months. The overall median survival rate of radical resection was 72/77, higher than the rate of palliative group (12/17) or transplantation group (17/21), which was also statistically significant (*P* < .01).

In our study, we believe in that all stages of hepatic alveolar echinococcosis should take active surgical interventions, and radical hepatic resection should be considered as the first-choice treatment for early stage of alveolar echinococcosis, while palliative surgery is still helpful to relieve symptoms and improve the life quality for advanced patients. Liver transplantation might also be an alternative option for the late-stage hepatic alveolar echinococcosis.

## 1 Introduction

Hepatic alveolar echinococcosis is also known as alveolar hydatid disease of liver. It is a type of parasitic zoonoses of tapeworms the Echinococcus can parasitize in diverse visceral organs of human beings such as liver, lung, spleen, and brain. Moreover, the primary lesion of nearly all the cases of human alveolar echinococcosis occurs in liver.^[1–4]^ Both disability and mortality rates are high in hepatic alveolar echinococcosis with the 10-year mortality rate of 90% as reported.^[5,6]^ Although hepatic alveolar echinococcosis is regarded as a benign disease; however, it is similar to the malignant tumor in terms of invasiveness known as the so-called insect carcinoma and secondary carcinoma’.^[7]^ This disease has been reported all around the world, especially in the pasturing areas, including Central Europe, Western Europe, Eastern Europe, northern North America, as well as the western part of China. Currently, the main treatment of hepatic alveolar echinococcosis is radical hepatic resection. Therefore, in our study, we compared several common surgical procedures aiming to provide the foundation for prioritizing therapeutic treatment.

## 2 Material and methods

This study was approved by the West China Hospital Ethics Committee and was performed in accordance with the ethical guidelines of the Declaration of Helsinki.

### 2.1 Patients

From January 2004 to June 2016, we collected the clinical data from 115 patients who received surgical treatment in West China Hospital. Among them, there were 51 male and 64 female patients. Their ages ranged from 6 to 82, with an average age of 47.8 ± 13.3 years. Around 115 patients were divided into 3 groups: 77 cases underwent radical hepatic resection (group A, n = 77); 17 cases underwent palliative resection (group B, n = 17), and 21 cases underwent liver transplantation (group C, n = 21) with 12 cases of orthotopic liver transplantation and 9 cases of liver autotransplantation (Table 1).

**Table 1:**
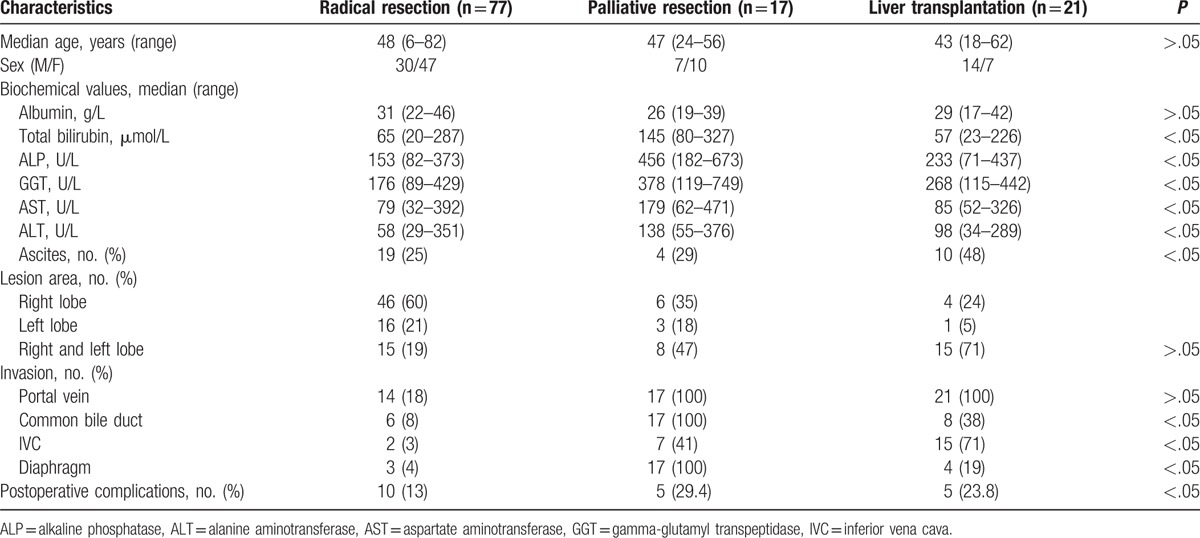
Baseline characteristics of 115 patients with hepatic alveolar echinococcosis.

The inclusion criteria were: signs and symptoms of alveolar echinococcosis with positive serological examination; computed tomography (CT) or magnetic resonance imaging (MRI) suggested hepatic lesions; and pathologically diagnosedwith alveolar echinococcosis postoperation. Those without valid clinical data or whole period of follow-up were excluded.^[8,9]^

### 2.2 Preoperative assessment

We ruled out diseases of heart, brain, lung and kidney, etc., as well as contraindications for surgery. Assessment of hepatic reserve function: all patients must be in Child–Pugh A class with indocyanine green (ICG) clearance at 15 minutes less than 10%. CT/MRI was used to analyze the location, size, number of the lesions, and their relationship with peripheral blood vessels. In addition, the total liver volume, potential residual liver volume after liver resection, the hepatic vascular, and biliary systems (e.g., the pattern of hepatic veins that drain into the inferior vena cava and whether there exist variations or drainage areas), as well as the anatomy features and potential variations of portal vein branches and the left/right hepatic ducts confluence area, etc., were all subjected to accurate evaluation. The surgical approach was decided accordingly.

### 2.3 Surgical approach

#### 2.3.1 Radical hepatectomy of hepatic alveolar echinococcosis

**Figure 1:**
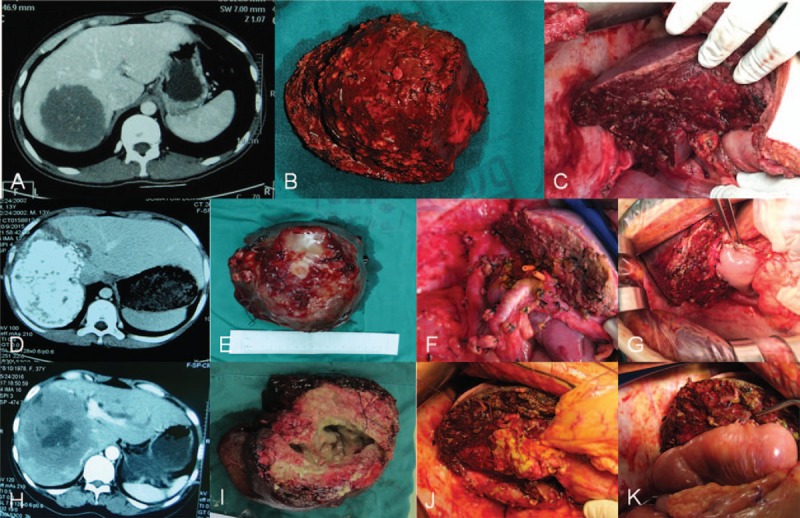
Simple radical hepatic resection (A–F): (A) CT showed the mass was in right liver and the first or the second hepatic hilum was not eroded; (B) resected specimen; (C) hepatic alveolar echinococcosis was underwent radical resection; Difficult radical hepatic resection (D–G): (D) CT showed the mass was in right liver and the first or the second hepatic hilum was eroded; (E) resected specimen; (F) hepatic alveolar echinococcosis was underwent radical resection; (G) Roux-en-Y hepaticojejunostomy was carried out. Palliative resection (H–K) (H) CT showed the hydatid lesion pressured and eroded the first hepatic hilum and left biliary ducts were obvious obstruction; (I) the gradual removal of hepatic alveolar echinococcosis; (J) hepatic alveolar echinococcosis was underwent palliative resection and revealed that the left side of bile duct dilatation; (K) Roux-en-Y hepaticojejunostomy was carried out.

With general anesthesia, we entered the abdominal cavity through right subcostal incision, and then determined the feasibility of radical hepatectomy of the lesion by intraoperative ultrasonography which could help to identify the location, size, and number of the lesions. Surgery margin was 1 cm away from the mass of the radical resection.^[10]^ Hepatic resection by cavitron ultrasonic aspiration (CUSA) could fully expose the small branches of the hepatic vein or glisson system, when the diameter of blood vessel or bile duct was greater than 2 mm, they would be ligated and cut off (Fig. 1A–C). In addition, patients with severe infiltration of hilar bile duct should receive choledochojejunostomy. For our data, Roux-en-Y hepaticojejunostomy was performed on 4 patients because of their biliary obstruction (Fig. 1D–F and J).

#### 2.3.2 Palliative hepatectomy

The major principles of palliative resection should be to relieve symptoms and prolong the life of patients.^[11]^ After general anesthesia, we took the right costal margin incision into the abdominal cavity, and then applied the intraoperative ultrasonography to confirm the location, size and numbers of the lesion. We would choose palliative hepatectomy if the intraoperative ultrasonography found that the first and second hepatic portal areas are severely infiltrated so that the radical hepatectomy cannot be performed on the patient. The method of liver resection in palliative hepatectomy was the same as that for radical resection. For the patients with biliary obstruction, we would remove lesions from the first hepatic hilum as much as possible to expose the dilated bile duct in the hepatic hilar region, which was required for Roux-en-Y hepaticojejunostomy, The Roux-en-Y hepaticojejunostomy was carried out successfully on 15 patients (Fig. 1H–K).

#### 2.3.3 Liver transplantation (LT)

Orthotopic liver transplantation could be performed on patients whose lesion is impossible to be removed by for the routine radical resection as well as when patients have no extra-hepatic metastases or the BDD (brain-dead donors)/DCD (donation after cardiac death) was available.

#### 2.3.4 Liver autotransplantation

For some patients with opportunities of vascular reconstruction for the first and second hepatic portal, we could cut the entire liver and then implant artificial inferior vena cava for them. Next, we removed the lesions in vitro, implanted the residual normal liver into the patient and reconstructed the major blood vessel and biliary tract, which could help achieving radical goals. If extra-hepatic metastases were found in patients, liver autotransplantation should not be performed.

### 2.4 Surgical treatment

In this group, 77 cases underwent the radical hepatic resection; 17 cases underwent palliative resection mainly due to the large size of the lesion combined with distal metastasis and invading porta hepatis, common bile duct, vena cava, diaphragm, or retroperitoneal region. Additionally, 21 patients cases underwent orthotopic liver transplantation (n = 12) or autotransplantation (n = 9) according to different donor sources.

Among 77 cases with radical surgery, 29 underwent right hemihepatectomy, 13 underwent right hepatic trisegmentectomies, 7 underwent left hemihepatectomy, 7 underwent left hepatic trisegmentectomies, 3 underwent left hepatic lobe resection, and 18 underwent segmental hepatectomy. Four patients underwent cholangioenterostomy because of biliary obstruction; 3 cases had combination of diaphragmatic muscle resection. Additionally, 17 cases underwent palliative resection, 6 had right hemihepatectomy, 3 had left hepatic lobe resection, 5 had right hepatic trisegmentectomies, and 3 had left hepatic trisegmentectomies. Roux-en-Y hepaticojejunostomy was carried out successfully in 15 patients; 21 cases underwent liver transplantation, including 12 cases with orthotopic liver transplantation using the liver of brain-dead donor. Another 9 cases of liver autotransplantation had their entire diseased liver removed. The inferior vena cava was replaced by artificial blood vessels and the remaining normal liver tissue was transplanted into patients with anastomosis of hepatic artery and the portal vein.

### 2.5 Postoperative treatment

Patients were given albendazole (200 mg tablets, 15 mg/(kg d), per os, bid) for more than 6 to 12 months continuously. Re-examinations were carried out 1, 3, and 12 months after surgery, including ultrasound, CT/MRI, liver function, routine blood test, antibody of hydatid disease. The follow-up re-examination will be performed once a year.

### 2.6 Statistical approach

Statistical analysis was performed using SAS 9.2 software. Categorical data were presented as ratio, analyzed via Pearson chi-squared (χ^2^) test; measurement data were presented as standard deviation of sample means (** ± *s*). Variance analysis was used for numeric continuous variables, and Kruskal Wallis was used for discrete data and non-normal distribution data. Kaplan–Meier was used for survival analysis. Statistical significance was defined as *P < .*05.

## 3 Results

### 3.1 Complications

No patients died during the operation. In the radical resection group, 10 (10/77, 13.0%) cases had complications. Among them, 2 patients have hypoproteinemia with abdominal cavity effusion; 3 patients have bile leakage; 4 patients have respiratory tract infection with pleural effusion, and 1 has incision infection. In palliative resection group, 5 (5/17, 29.4%) cases had complications included 3 cases bile leakage with abdominal cavity infection, 2 cases had respiratory tract infection with pleural and abdominal effusion. All patients in the above 2 groups, were cured by nonsurgical methods.

In the liver-transplant group, 5 cases (5/21, 23.8%) had complications, including 1 case with respiratory infection, 1 case with acute rejection reaction, and 1 case with hepatic encephalopathy and coagulation disorder, 2 cases with abdominal bleeding. Four patients died and only 1 patient with respiratory infection survived due to the anti-infection treatment. Thus, the incidence of complications in radical resection group was significantly lower than the palliative resection or liver transplantation group (*P < .*05).

### 3.2 Postoperative follow-up

The follow-up period ranged from 1 to 72 months, with average of 36 months. During the period, A group: 2 cases died from new lesions of hydatid after 36 months and 48 months; B group: 5 cases died from hydatid recurrence after 13, 18, 24, 24, and 36 months; and C group: 4 cases died from severe complications after liver transplantation, including 3 patients died within 6 months of allotransplantation. Among the 3 patients, 2 had abdominal bleeding; 1 had multiple organ failure after hepatic encephalopathy with coagulation disorder and 1 case died of infection after 2 years. The other 17 patients achieved long-term survival after liver transplantation.

**Figure 2:**
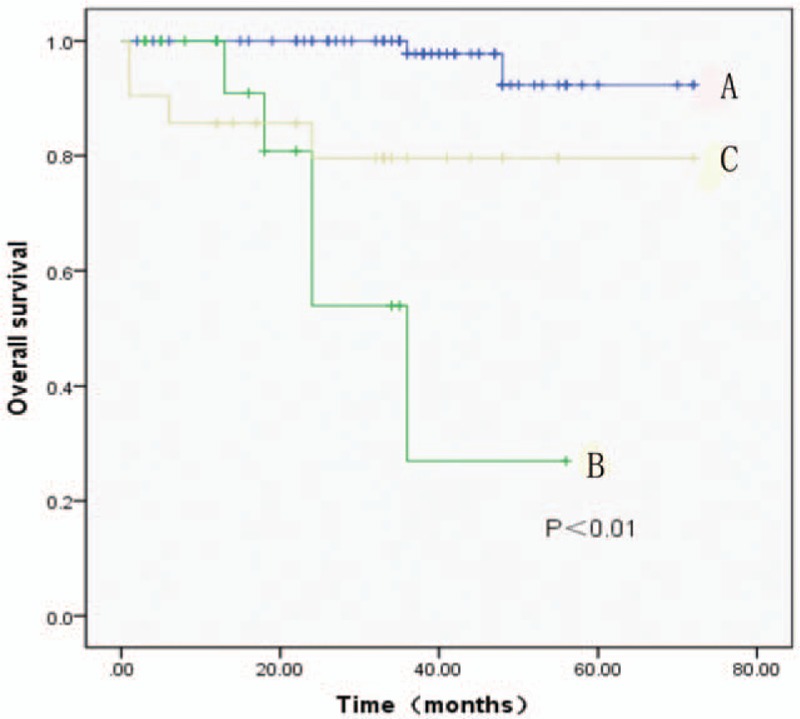
Kaplan–Meier estimated survival curves based on different surgical therapies with radical resection group (A), palliative resection group (B), liver transplantation group (C). The lifetime is obviously longer in A than B or C (*P* < .01).

The overall survival rate of group A was 97% with clinical cure in 75 patients. The overall survival rate of group B was 70.6% with 12 patients survived. For group C, the overall survival rate was 81%. Statistical significance (*P* < .01) was shown by comparing group A and group B as well as group A and group C regarding to the survival time, which indicated that the lifetime is significantly longer in Group A (Fig. 2).

## 4 Discussion

With continuous attempt to treat hepatic alveolar echinococcosis, the therapeutic outcome has been tremendously improved. Until now, surgical procedure is still the first choice. Therefore, we collected the clinical data and conducted this study to further evaluate the outcomes of radical hepatic resection, palliative resection, and liver transplantation, which are the 3 major surgical procedures.^[12]^

Radical hepatic resection is the most ideal procedure to treat hepatic alveolar echinococcosis, mainly because of its thorough resection and low recurrence rate.^[2,13,14]^ However, the complications rate is quite high due to the heavy surgical trauma. In our study, the complication rate is relatively high (13.0%); however, the recurrence rate is low (2.6%) without any death case in the radical hepatic resection group during operation. Previous study showed that, the survival rate for patients with en bloc resection with negative margin was 100% after a 300-month follow-up period.^[4]^ Therefore, for hepatic alveolar echinococcosis, radical resection should be considered as the first choice if the condition allows.^[2]^ In addition, for localized lesions without involvement of the first porta hepatis, portal vein, or the common hepatic artery, the remaining volume of liver is enough to meet the metabolic needs.

Considering the occult onset of hepatic alveolar echinococcosis and the difficulties of early diagnosis, most of the patients could be asymptomatic in the early stage, which means they may have lost the possibility to receive radical resection due to distal metastasis of alveolar echinococcosis.^[15]^ Therefore, the principle for palliative surgery with biliary reconstruction in the present study provided patients the surgery opportunity, relieved symptom and prolonged life. By comparing with the radical surgery, palliative resection with residual lesions result in higher complication rate (29.4%) due to secondary biliary fistula, further abdominal infection and the potential pleural effusion with respiratory infection by the necrosis and exfoliation of the residual lesions.^[16,17]^ However, palliative resection with biliary reconstruction can significantly increase the 1-year survival rate and achieve a better life quality for patients with end stage alveolar echinococcosis disease. The economic burden is extremely small since patients recover quickly without severe complications. Nevertheless, palliative resection is great efficient method regarding to relieving the symptoms and slowing the disease progression.^[18]^

As the therapeutic approach for end-stage liver disease, liver transplantation tends to be mature and capable of providing the opportunities on late-stage hepatic alveolar echinococcosis.^[19]^ However, the feasibility of liver transplantation is limited by its high cost, the shortage of donor organs, the demand of high surgical technique and the need for immunosuppression.^[20,21]^ Moreover, liver autotransplantation which removed the entire diseased liver with replacement of the inferior vena cava by artificial blood vessels and then transplanted the remaining normal liver tissue itself with difficult anastomosis of hepatic artery and the portal vein, bring in troublesome technical issues.^[22]^ In our study, 3 cases died in 6 months after autotransplantation, which was a high mortality rate during the perioperative period. Additionally, the economic burden of operation and immunosuppression is extremely high: the average cost of the 77 patients receiving radical surgery or the 17 patients receiving palliative resection was about 6000 to 7000 USD during the perioperative period, while liver transplantation could be tenfold higher which is not affordable for many patients. Furthermore, extra-hepatic metastases were usually found in many end stage alveolar echinococcosis patients.^[23]^ It is obvious that only few patients are suitable for liver transplantation and autotransplantation. So, liver transplantation and autotransplantation should not be considered as routine methods for hepatic alveolar echinococcosis.

This study has several limitations that should be addressed. First, this is a retrospective study from a single-institution experience. The number of patients enrolled may be not sufficient enough and the sample size of 3 groups is different. The impact of various treatments related to outcome could not be fully evaluated and the follow-up duration of the study may be not long enough. Second, all the data were collected through the medical records and follow-up recall bias and selection bias possibly exists. Therefore, large cases of randomized controlled long-term observation researches are expected in the future.

## 5 Conclusion

In our study, we believe in that all stages of hepatic alveolar echinococcosis should take active surgical interventions, and radical hepatic resection should be considered as the first choice in treatment for early stage of alveolar echinococcosis. Additionally, palliative surgery was still helpful to relieve symptoms, improve quality of life, and delay the disease progression for advanced patients who had already lost the opportunity to receive radical resection. Liver transplantation might also be an alternative option for late-stage hepatic alveolar echinococcosis, but it has high surgical risk and indications for liver transplantation should be strictly controlled.

## Acknowledgments

The authors would like to thank the Liver Transplantation Center in West China Hospital of Sichuan University, which provided the research platform as well as technical support. We thank Doctor Zhe-yu Chen for the full skillful assistance in the hydatid surgery and thank for his revision.

This research did not receive any specific grant from funding agencies in the public, commercial, or not-for-profit sectors.
